# Contrasting Climate Change Responses of the Rare Moss Genus *Tetrastichium* (Mitt.) Cardot Across Macaronesia: Implications for Persistence and Conservation

**DOI:** 10.3390/plants15182742

**Published:** 2026-09-08

**Authors:** Ruymán David Cedrés-Perdomo, Guillermo Santos, Anabela Martins, Juan José García-Alvarado

**Affiliations:** 1 Departamento de Botánica, Ecología y Fisiología Vegetal, Universidad de La Laguna, 38206 La Laguna, Spain; jjgarciaalvarado@gmail.com; 2 Departamento de Biodiversidad, Ecología y Evolución, Facultad de Ciencias Biológicas, Universidad Complutense de Madrid, 28040 Madrid, Spain; gsanto02@ucm.es; 3 CE3C—Centre for Ecology, Evolution and Environmental Changes & CHANGE—Global Change and Sustainability Institute, Faculdade de Ciências, Universidade de Lisboa, 1749-016 Lisbon, Portugal; anabelamartins323@gmail.com

**Keywords:** bryophytes, climatic suitability, species-distribution models, oceanic islands

## Abstract

Climate change poses a threat to bryophytes with restricted distributions and strong dependence on humid habitats. We assessed the climatic suitability of the moss genus *Tetrastichium* across the Azores, Madeira, and the Canary Islands. We compiled 90 records each for *Tetrastichium fontanum* and *Tetrastichium virens* and fitted Boosted Regression Tree models using WorldClim 2.1 bioclimatic predictors. Future suitability was projected for 2061–2080 and 2081–2100 using two global climate models under SSP3-7.0 and SSP5-8.5. Model performance was good (AUC = 0.88, sTSS = 0.81, and sensitivity ≥ 0.94). Under current and future conditions, suitable areas were unevenly distributed among archipelagos. Climatically suitable areas generally increased in the Azores but contracted substantially in Madeira and the Canary Islands. Under UKESM1-0-LL, no suitable areas in the Canary Islands remained above the climatic-suitability threshold by the end of the century. Spatial overlap between current and future predictions declined under stronger climate forcing and longer time horizons, being consistently higher for *T. virens*. These findings reveal the high sensitivity of *Tetrastichium* to climate change, identifying Madeira and the Canary Islands as areas of conservation concern. Protecting extant populations and environmentally buffered habitats will be essential for the genus’s persistence under future climates.

## 1. Introduction

Species distributions are dynamic outcomes of interactions among environmental conditions, dispersal capacity, biotic interactions, evolutionary history, and anthropogenic pressures, rather than fixed geographical boundaries [1,2,3]. Among these drivers, climate change is expected to profoundly reshape biodiversity patterns by altering temperature and precipitation regimes, increasing the frequency of climatic extremes, and modifying the environmental conditions required for species persistence [4]. Anticipating these changes has become a major challenge in conservation biology, particularly for species with narrow ecological requirements, fragmented distributions, and limited occurrence data [5,6,7]. Nevertheless, conservation priorities should not be inferred solely from geographical range size, as species differ considerably in their exposure to habitat degradation, anthropogenic pressures, and climate change [8]. In this context, species-distribution models (SDMs) are widely used to identify climatically suitable areas, explore potential distributional shifts, and support conservation planning. However, SDMs represent potential environmental suitability rather than realized distributions, and their predictive performance depends on the quality of occurrence data, the selection of environmental predictors, and the explicit consideration of uncertainty [3,7,9].

These challenges are particularly relevant for rare and poorly sampled organisms, whose apparent rarity may reflect genuine ecological restriction, low detectability, historical under-collection, or uneven survey effort [6,10]. Spatial sampling biases toward accessible localities, protected areas, and historically well-surveyed sites can distort inferred suitability patterns and conservation metrics, especially when narrow-ranged taxa are modeled using small occurrence datasets [11,12,13]. In bryophytes, recent studies have shown that informative predictions can still be obtained for rare species when occurrence records are taxonomically verified, spatially filtered, and analyzed with carefully calibrated modeling approaches [13,14,15]. For conservation purposes, SDMs should therefore be interpreted alongside ecological knowledge, habitat availability, and field validation, particularly when used to identify targets for future surveys [7,16].

Bryophytes are highly sensitive to climatic and microclimatic variation because their physiological activity depends strongly on external water availability, and their desiccation–rehydration dynamics are closely linked to humidity, temperature, and radiation regimes [17,18]. Despite their ecological importance, bryophytes remain relatively underrepresented in ecological research and conservation planning, although the application of SDMs to this group has increased substantially in recent years [19,20]. At broad spatial scales, their distributions are strongly influenced by regional climatic conditions, whereas local persistence depends on factors such as substrate availability, canopy cover, forest continuity, hydrology, topography, and microclimatic buffering [15,21,22,23]. These fine-scale environmental characteristics are often poorly represented in global climatic datasets, particularly in humid forests, ravines, stream margins, and other environmentally buffered habitats [23,24,25,26]. Nevertheless, macroclimatic SDMs remain useful for evaluating broad-scale suitability patterns, provided that their predictions are interpreted cautiously and in conjunction with ecological and field-based information [16,27,28,29,30].

Macaronesia is an exceptional natural laboratory for studying bryophyte biogeography and the effects of climate change. Its oceanic archipelagos combine geographic isolation, pronounced elevational gradients, and remarkable environmental heterogeneity over relatively small spatial scales [27,31,32,33]. Variations in altitude, geological history, trade-wind exposure, and precipitation regimes create a wide array of habitats, supporting one of Europe’s richest bryophyte floras [27,34]. In particular, laurel forests, heath forests, ravines, springs, and other humid habitats provide persistently moist, thermally buffered conditions and may therefore serve as refugia for moisture-dependent bryophytes [24,35,36]. Recent studies suggest that these environmentally buffered habitats may become increasingly important under future climate change, although the extent to which they will continue to maintain suitable conditions remains uncertain [37]. Understanding the spatial distribution of climatic suitability across these islands is therefore essential for identifying conservation priorities and supporting the long-term protection of Macaronesian bryophyte diversity [24].

The moss genus *Tetrastichium* (Mitt.) Cardot belongs to the family Leucomiaceae and is represented in the Ibero-Macaronesian region by two species, *Tetrastichium fontanum* (Mitt.) Cardot and *Tetrastichium virens* (Cardot) S.P.Churchill [38,39]. Both species have restricted, geographically discontinuous distributions, with records concentrated in Macaronesia and the southwestern Iberian Peninsula [40,41,42,43,44]. Their conservation significance is underscored by their inclusion in the European Red List of Bryophytes, where *T. fontanum* is classified as Vulnerable (VU) and *T. virens* as Near Threatened (NT) [34]. Both species are strongly associated with permanently humid, shaded habitats, particularly laurel forests and other environmentally buffered microhabitats that maintain high humidity and reduced thermal variability, such as ravines, springs, cave entrances, and lava tubes [35,36,43,44,45,46]. This close association with humid refugial habitats, together with their fragmented distribution and conservation status, makes *Tetrastichium* an excellent model for investigating the role of climatic refugia in the persistence of rare bryophytes under climate change [37].

Despite their conservation significance, the climatic suitability of *Tetrastichium* species has not yet been comprehensively evaluated across Macaronesia. This knowledge gap limits our understanding of the environmental drivers of their fragmented distribution and constrains conservation planning under ongoing climate change [7,47]. Here, we model the potential climatic suitability of *T. fontanum* and *T. virens* across Macaronesia using taxonomically verified, spatially filtered occurrence records within a species-distribution modeling framework. Specifically, we aim to characterize the climatic suitability of *T. fontanum* and *T. virens* across Macaronesia, identify climatically suitable areas that may be priority targets for future field surveys, and explore the conservation implications of the predicted suitability patterns. We hypothesize that climatically suitable areas for both species will be concentrated in humid regions of Macaronesia, particularly in laurel forests and other environmentally buffered habitats that may act as climatic refugia [24,36,37,46]. By combining species-distribution modeling with ecological knowledge, this study provides a spatial framework for identifying climatically suitable areas, guiding future field surveys, and supporting conservation planning [5,7,16,48].

## 2. Results

### 2.1. Model Performance and Predictor Importance

**Figure 1:**
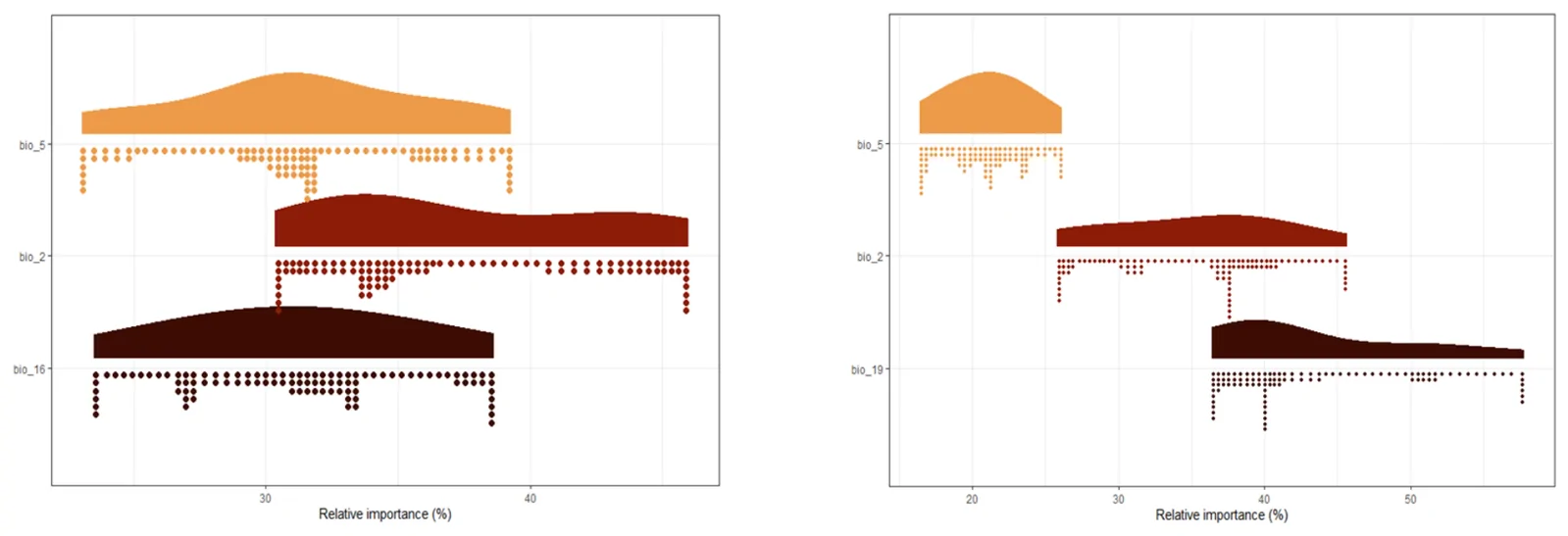
Relative contribution (%) of the selected bioclimatic variables to the Boosted Regression Tree (BRT) models for *Tetrastichium fontanum* and *Tetrastichium virens*. Predictor importance is expressed as the percentage contribution of each variable to the final models. The stacked circles form dot histograms showing the frequency distribution of the importance values obtained for each predictor, while the shaded curves represent their smoothed distributions. BIO2 = Mean Diurnal Range; BIO5 = Maximum Temperature of the Warmest Month; BIO16 = Precipitation of the Wettest Quarter; BIO19 = Precipitation of the Coldest Quarter.

The relative importance of the selected climatic variables in the species-distribution models differed between species (Figure 1). In the *Tetrastichium fontanum* models, Mean Diurnal Range (BIO2) was the most important predictor, while Maximum Temperature of Warmest Month (BIO5) and Precipitation of Wettest Quarter (BIO16) made similarly substantial contributions. Each of these three variables individually accounted for more than 30% of the total contribution. This pattern was not observed in the *Tetrastichium virens* models, in which Precipitation of Coldest Quarter (BIO19) was the most influential variable, contributing approximately 40%, followed by BIO2 and BIO5, each contributing approximately 35% and 20%, respectively. Response curves showed that climatic suitability for both species was generally associated with smaller mean diurnal temperature ranges and lower maximum temperatures during the warmest month. Suitability for *T. virens* increased with precipitation during the coldest quarter, whereas *T. fontanum* exhibited a non-monotonic response to precipitation during the wettest quarter.

Overall, the models for both species showed good predictive performance (Table 1). Both the standardized True Skill Statistic (sTSS) and the area under the receiver operating characteristic curve (AUC-ROC) exceeded 0.8, and sensitivity values were greater than 0.9. The Boyce Index also indicated good model performance, with values averaging approximately 0.7 ± 0.2. This indicates that the models generally discriminated well between presences and pseudo-absences across the predicted suitability gradient. Positive values show that species occurrences were more frequent than expected by chance in areas predicted to be highly suitable. However, the observed variability among replicates suggests that model performance ranged from moderate to very good, indicating some differences in predictive stability across model runs. Finally, Nagelkerke’s pseudo-R^2^ values were consistently above 0.5 and reached approximately 0.6 in some model runs, indicating that the fitted models showed a substantial improvement in model fit relative to the corresponding null models.

### 2.2. Climatic Suitability of Tetrastichium fontanum

**Figure 2:**
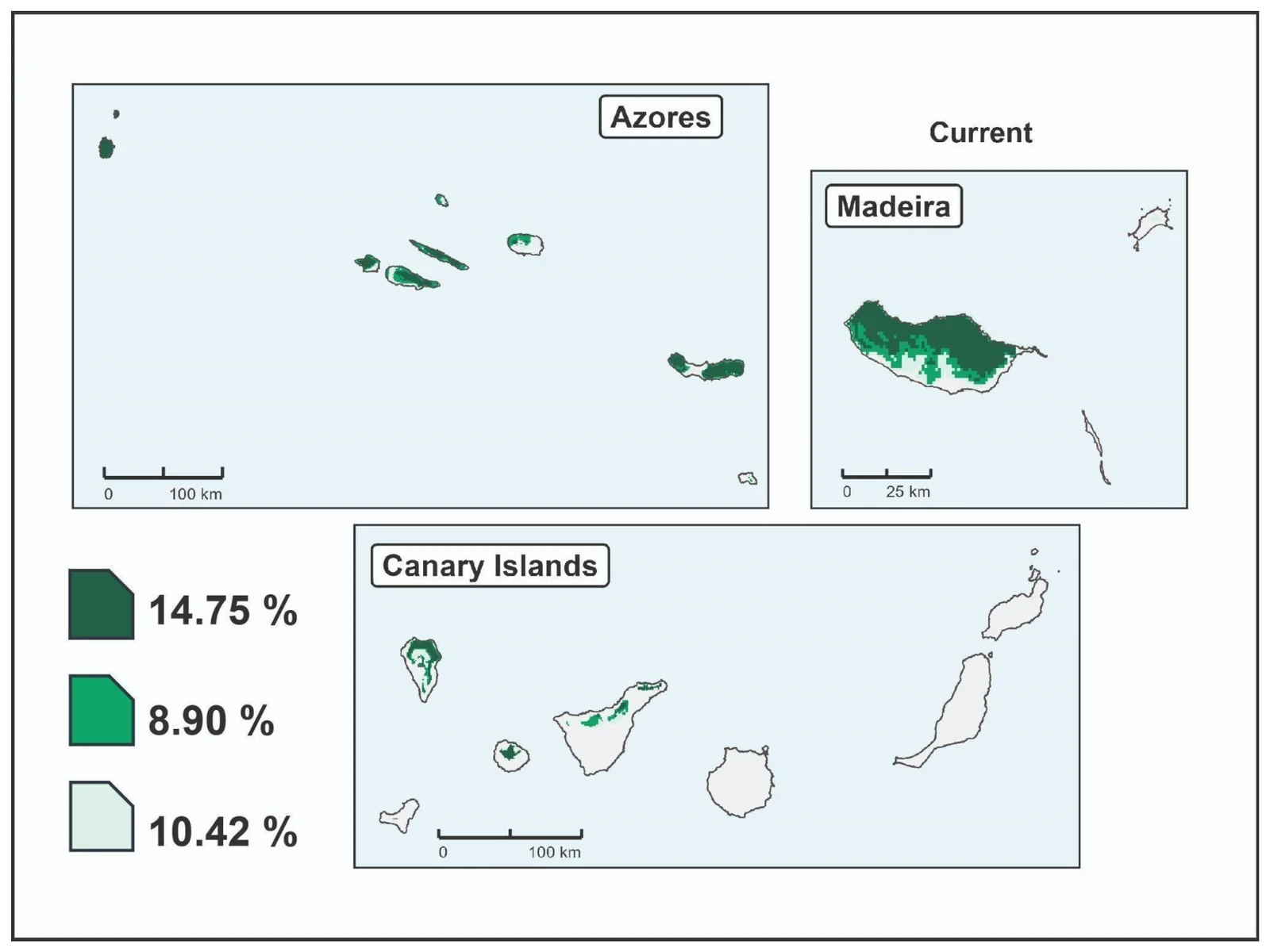
Current potential climatic suitability for *Tetrastichium fontanum* across the Azores, Madeira, and the Canary Islands predicted by the Boosted Regression Trees model. Suitability values were classified as low (0.25–0.50; light green), moderate (0.50–0.75; green), and high (0.75–1.00; dark green); areas with values below 0.25 were considered unsuitable. Percentages indicate the proportion of the total area of the study region assigned to each suitability class.

Current potential climatic suitability for *Tetrastichium fontanum* is shown in Figure 2. The species’ total potentially suitable area within Macaronesia accounts for 34.07% of the study region, comprising 14.75% high suitability, 8.90% moderate suitability, and 10.42% low suitability. However, this potential area is unevenly distributed among archipelagos and across suitability classes. A large proportion of the Azores and Madeira exhibits some degree of climatic suitability, whereas suitable areas in the Canary Islands are mainly restricted to the western islands of Tenerife, La Palma, La Gomera, and El Hierro.

**Figure 3:**
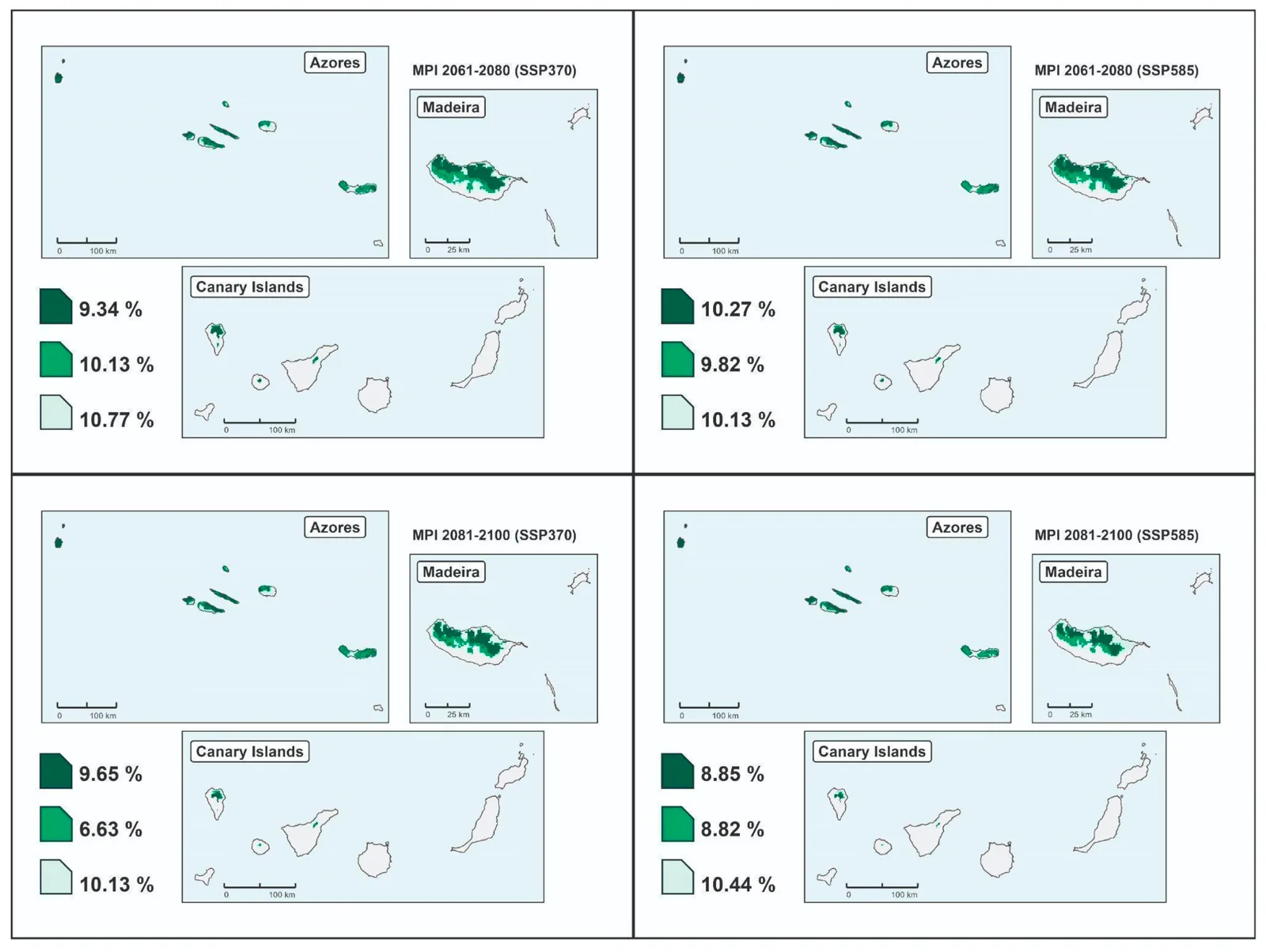
Projected climatic suitability for *Tetrastichium fontanum* across the Azores, Madeira, and the Canary Islands under the MPI-ESM1-2-HR global climate model for the SSP3-7.0 and SSP5-8.5 scenarios during the periods 2061–2080 and 2081–2100. Suitability values were classified as low (0.25–0.50, light green), moderate (0.50–0.75, green), and high (0.75–1.00, dark green); areas with values below 0.25 were considered unsuitable. Percentages indicate the proportion of the total terrestrial area of the study region assigned to each suitability class.

**Figure 4:**
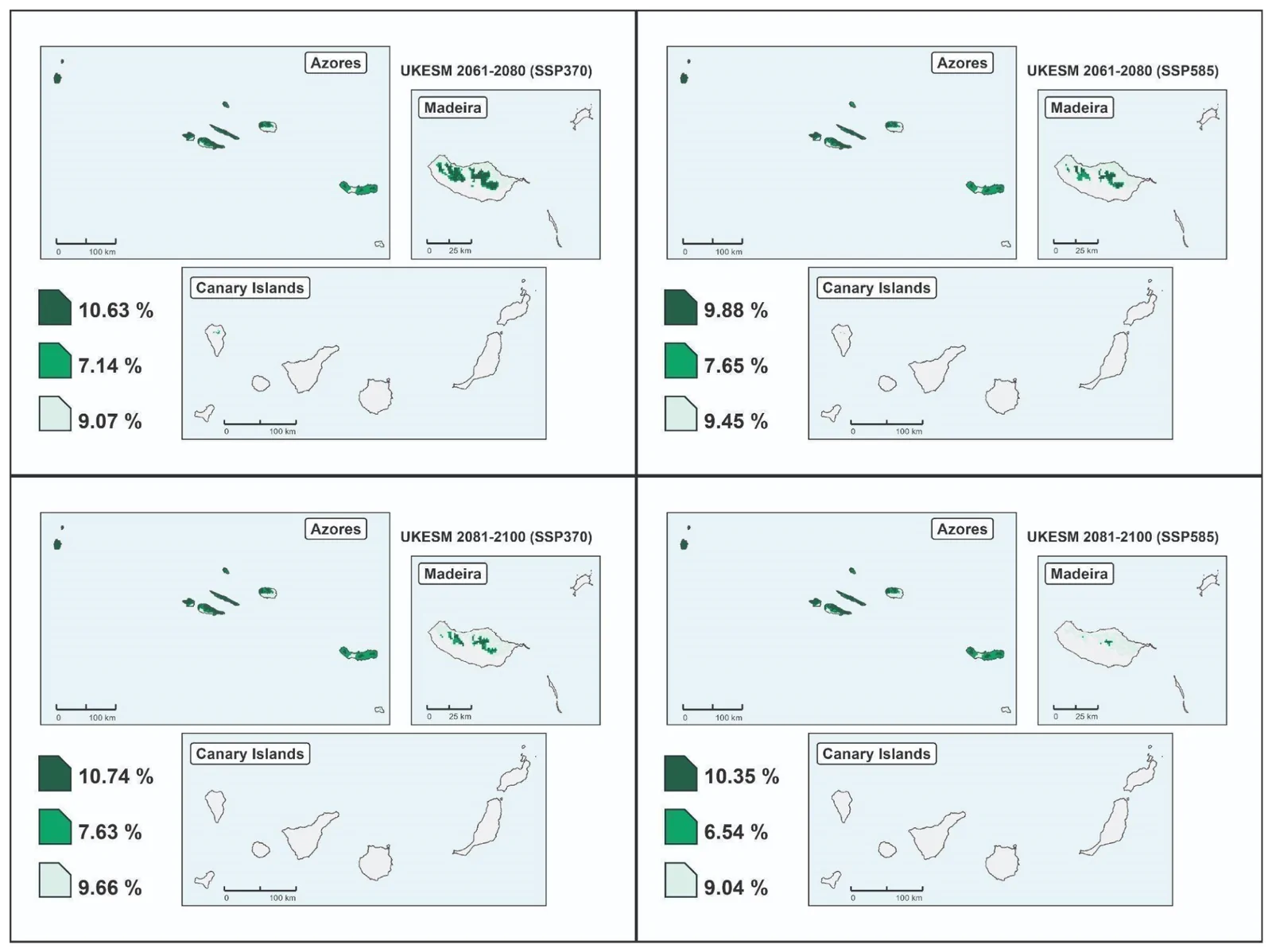
Projected climatic suitability for *Tetrastichium fontanum* across the Azores, Madeira, and the Canary Islands under the UKESM1-0-LL global climate model for the SSP3-7.0 and SSP5-8.5 scenarios during the periods 2061–2080 and 2081–2100. Suitability values were classified as low (0.25–0.50, light green), moderate (0.50–0.75, green), and high (0.75–1.00, dark green); areas with values below 0.25 were considered unsuitable. Percentages indicate the proportion of the total terrestrial area of the study region assigned to each suitability class.

The pattern observed under current climatic conditions, in which the low- and high-suitability classes are better represented than the moderate-suitability class, remained broadly consistent across future projections. Across the different time periods and emission pathways, climatically suitable areas declined in both Madeira and the Canary Islands (Figure 3 and Figure 4). Madeira exhibited the largest absolute reduction in the area classified as having high climatic suitability, with losses ranging from 17.6 percentage points under the MPI-ESM1-2-HR SSP3-7.0 scenario to 43.4 percentage points under the UKESM1-0-LL SSP5-8.5 scenario (Table 2; Figure 3 and Figure 4). In contrast, climatically suitable areas in the Canary Islands progressively contracted across all scenarios and disappeared entirely under the UKESM1-0-LL projections, although only a comparatively small area was classified as climatically suitable under current conditions.

### 2.3. Climatic Suitability of Tetrastichium virens

**Figure 5:**
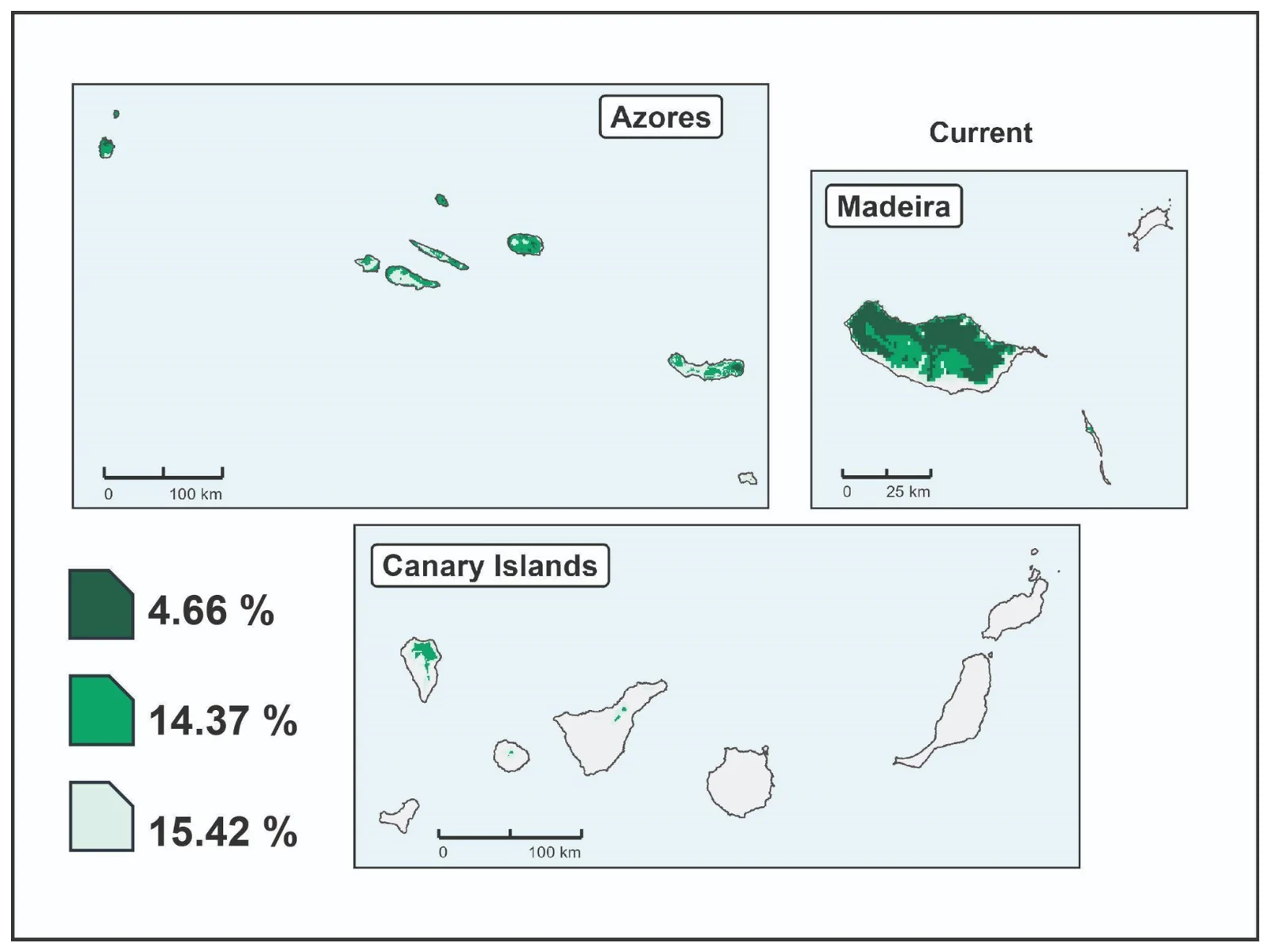
Current potential climatic suitability for *Tetrastichium virens* across the Azores, Madeira, and the Canary Islands predicted by the Boosted Regression Tree model. Suitability values were classified as low (0.25–0.50, light green), moderate (0.50–0.75, green), and high (0.75–1.00, dark green); areas with values below 0.25 were considered unsuitable. Percentages indicate the proportion of the total area of the study region assigned to each suitability class.

Current potential climatic suitability for *Tetrastichium virens* is shown in Figure 5. The species’ total potentially suitable area within Macaronesia accounts for 34.45% of the study region, comprising 4.66% high suitability, 14.37% moderate suitability, and 15.42% low suitability. However, this potential area is unevenly distributed among archipelagos and across suitability classes. A large proportion of the Azores and Madeira exhibits some degree of climatic suitability, whereas suitable areas in the Canary Islands are much more restricted, occurring mainly on La Palma, with smaller isolated areas on La Gomera and Tenerife.

**Figure 6:**
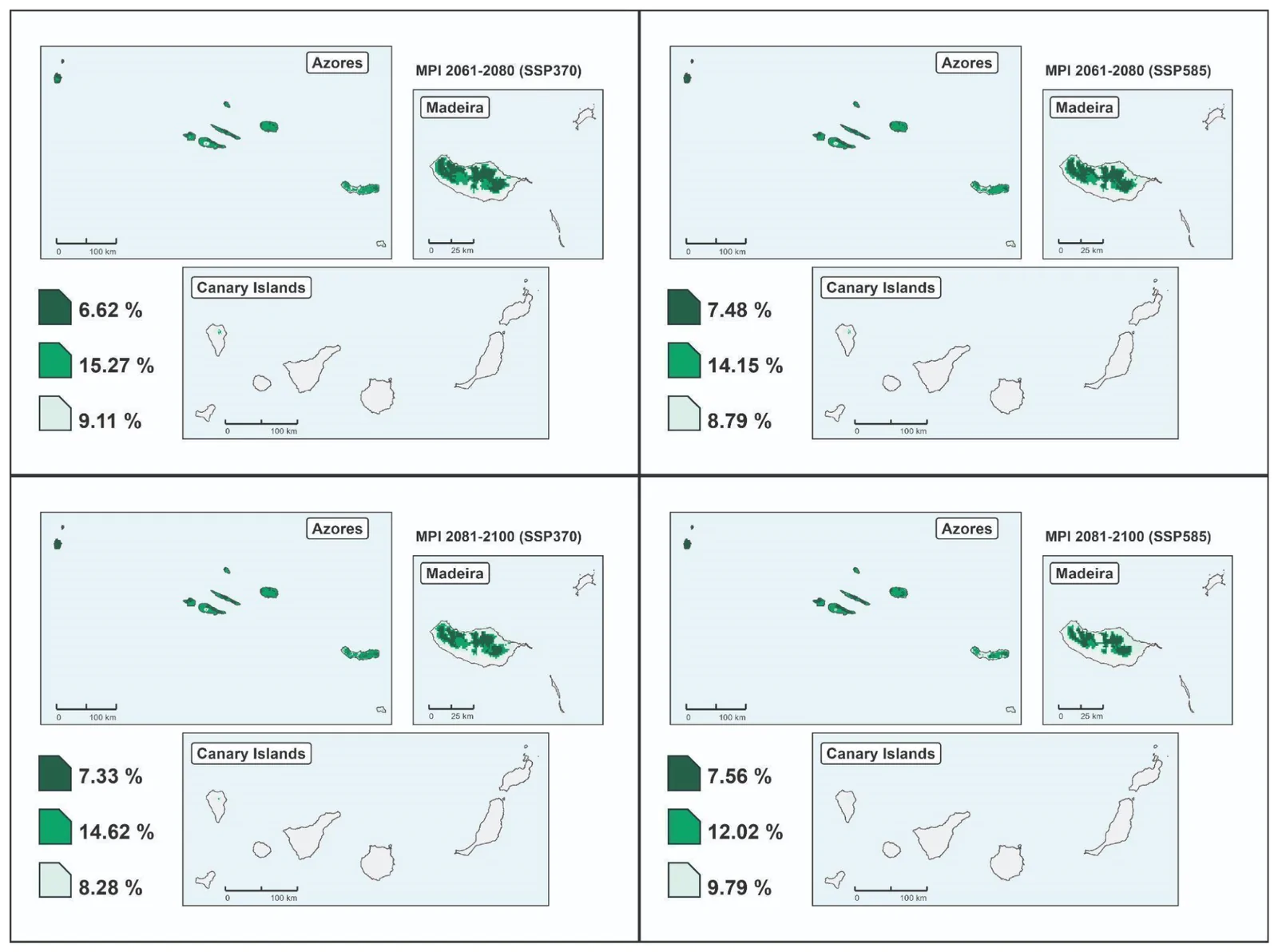
Projected climatic suitability for *Tetrastichium virens* across the Azores, Madeira, and the Canary Islands under the MPI-ESM1-2-HR global climate model for the SSP3-7.0 and SSP5-8.5 scenarios during the periods 2061–2080 and 2081–2100. Suitability values were classified as low (0.25–0.50, light green), moderate (0.50–0.75, green), and high (0.75–1.00, dark green); areas with values below 0.25 were considered unsuitable. Percentages indicate the proportion of the total terrestrial area of the study region assigned to each suitability class.

**Figure 7:**
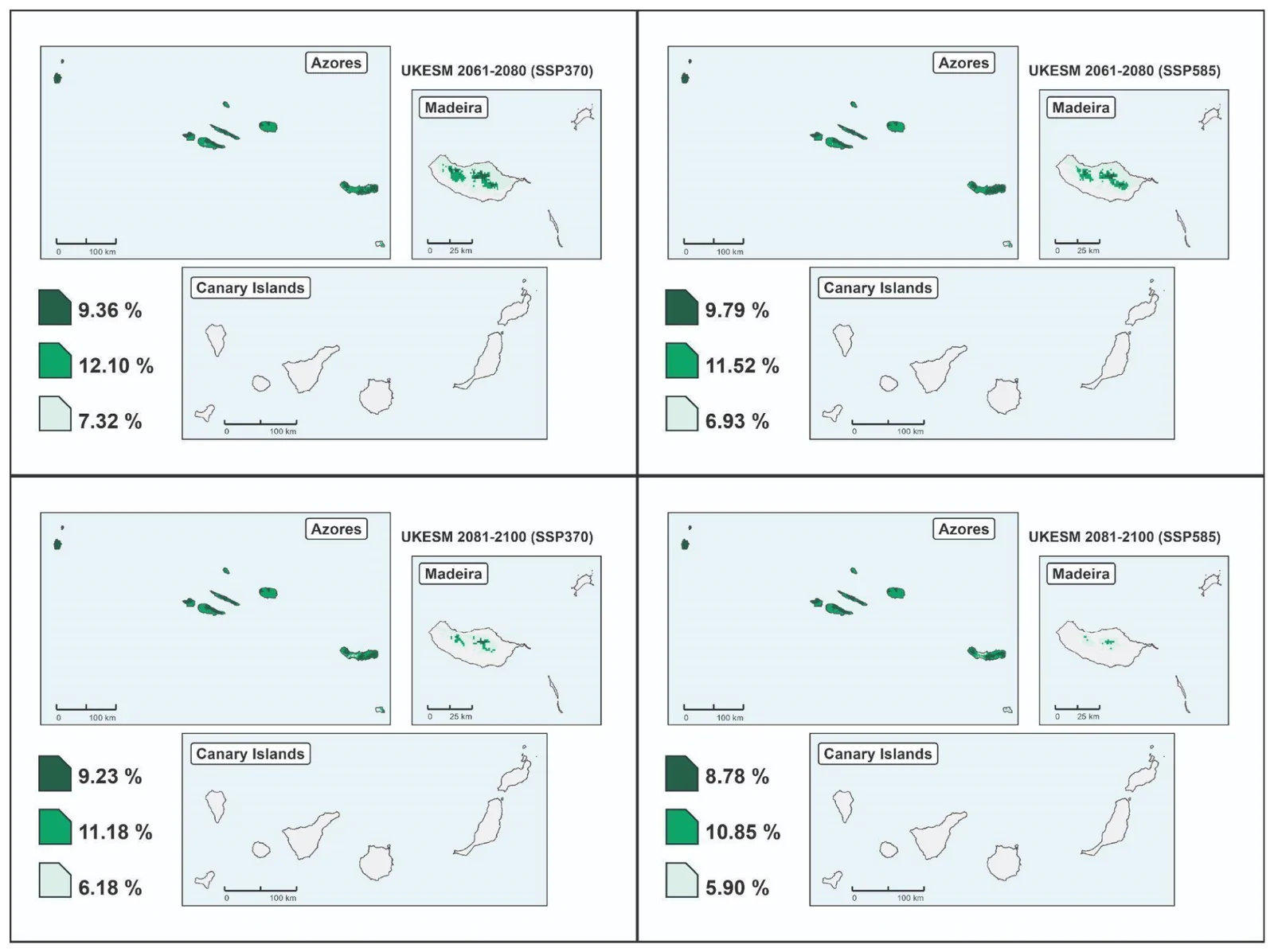
Projected climatic suitability for *Tetrastichium virens* across the Azores, Madeira, and the Canary Islands under the UKESM1-0-LL global climate model for the SSP3-7.0 and SSP5-8.5 scenarios during the periods 2061–2080 and 2081–2100. Suitability values were classified as low (0.25–0.50, light green), moderate (0.50–0.75, green), and high (0.75–1.00, dark green); areas with values below 0.25 were considered unsuitable. Percentages indicate the proportion of the total terrestrial area of the study region assigned to each suitability class.

Future projections show contrasting responses across the three archipelagos (Figure 6 and Figure 7). In the Azores, climatic suitability generally improved across both GCMs and emission pathways, with high-suitability areas increasing from 5.9% under current conditions to 18.1–24.8% under the MPI-ESM1-2-HR projections and 35.3–38.6% under UKESM1-0-LL (Table 3). By contrast, Madeira showed a consistent decline in climatically suitable area, with high-suitability areas decreasing from 39.5% at present to 17.3–26.3% under the MPI-ESM1-2-HR projections and nearly disappearing under the UKESM1-0-LL SSP5-8.5 scenario (Table 3). In the Canary Islands, climatically suitable areas, already highly restricted under current conditions, contracted further in all future projections and disappeared entirely under the UKESM1-0-LL projections by the end of the century. Overall, the projections identify the Azores as the most climatically favorable archipelago for *T. virens*, whereas Madeira and especially the Canary Islands are projected to experience substantial reductions in climatic suitability under future climate scenarios.

### 2.4. Changes in Climatic-Suitability Overlap

**Figure 8:**
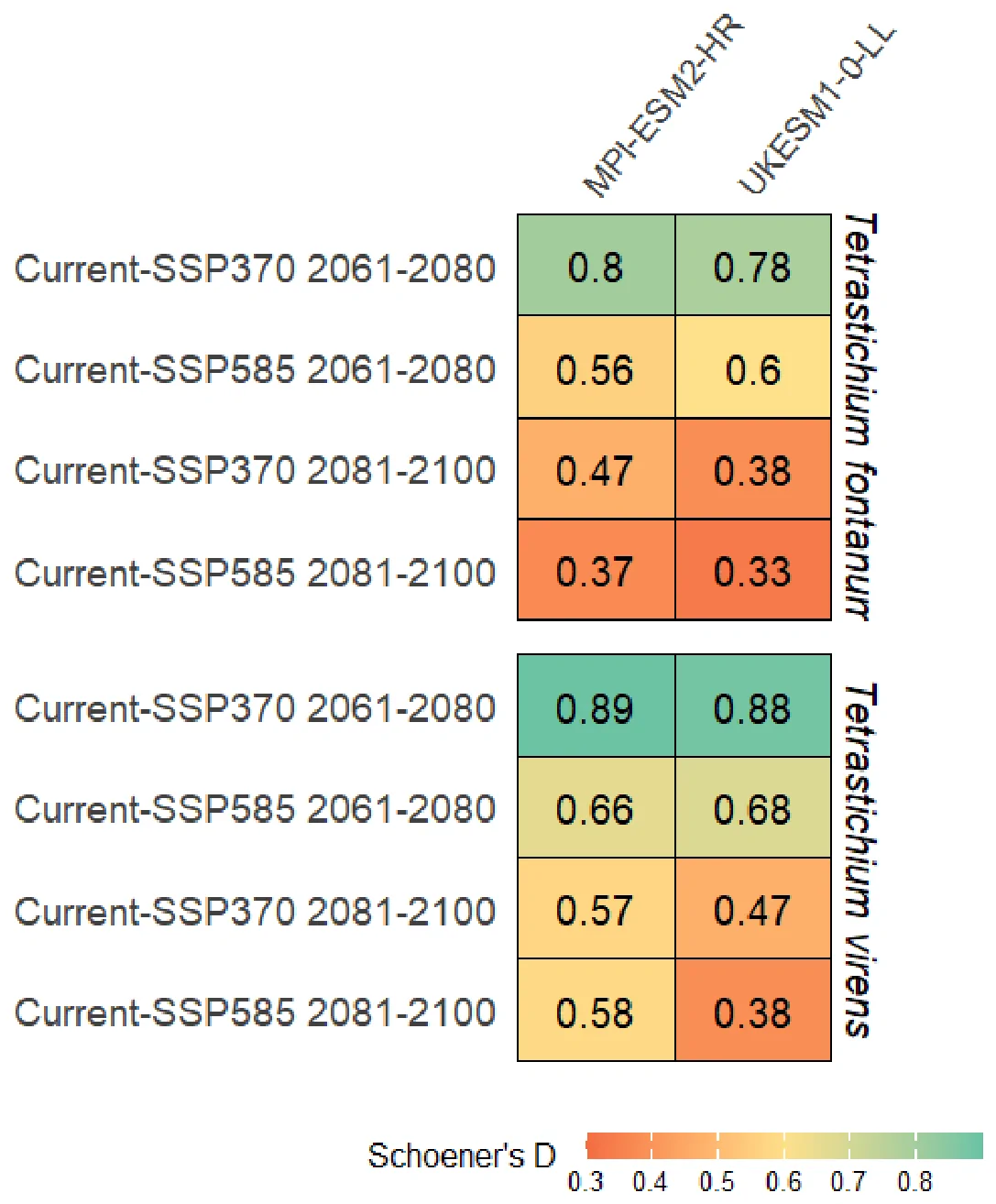
Schoener’s D values measuring the overlap between current and future climatic-suitability predictions for each species under different global climate models (GCMs), shared socioeconomic pathways (SSPs), and time periods.

Schoener’s D revealed a broadly consistent pattern across both species (Figure 8), with overlap generally decreasing under the higher-sensitivity GCM, the more severe emission pathway, and the longer projection horizon. Accordingly, overlap was generally higher for the 2061–2080 period, SSP3-7.0, and MPI-ESM1-2-HR, whereas the lowest values for both species occurred under UKESM1-0-LL, SSP5-8.5, and 2081–2100. Across all scenarios and GCMs, overlap was higher for *T. virens* than for *T. fontanum*.

## 3. Discussion

This study presents the first regional assessment of the climatic suitability of *Tetrastichium fontanum* and *T. virens* across Macaronesia. Despite their similar ecological requirements and association with humid, environmentally buffered habitats [43,44], the two species exhibited contrasting responses to projected climate change. Climatically suitable area increased in the Azores, although redistribution among suitability classes differed between species and GCMs, whereas both species showed substantial contractions in Madeira and the Canary Islands, particularly under UKESM1-0-LL and SSP5-8.5. These patterns are consistent with previous projections of severe but species-specific climate impacts on Macaronesian bryophytes [24,48] and scenario-dependent range dynamics in other mosses [49], reinforcing the need to consider variation among species, archipelagos, GCMs, and emission pathways when assessing conservation vulnerability [50].

Current climatic-suitability patterns broadly matched the published distributions of both species, with extensive suitable areas in the Azores and Madeira and more restricted areas in the western Canary Islands [43,44]. This pattern reflects broad Macaronesian climatic gradients and the influence of temperature and precipitation on bryophyte distributions [27,51,52]. Mean diurnal range and maximum temperature of the warmest month were important predictors for both species, whereas precipitation effects differed seasonally. The response curves indicated associations with smaller diurnal temperature ranges and lower maximum temperatures during the warmest month, consistent with the occurrence of both species in humid and thermally buffered environments. Predicted suitability for *T. virens* increased with precipitation during the coldest quarter, whereas *T. fontanum* showed a more complex, non-monotonic response to precipitation during the wettest quarter. These relationships are ecologically plausible for poikilohydric bryophytes [17,18], but should be interpreted as broad macroclimatic associations rather than as physiological thresholds or direct measurements of drought exposure, desiccation tolerance, or local microclimatic conditions [53].

Spatial overlap between current and future climatic-suitability patterns generally declined with longer time horizons and stronger climate forcing and varied among GCMs and SSPs, indicating substantial geographical reorganization and potential vulnerability to associated range shifts [54]. Model disagreement was greatest for *T. fontanum*, for which both the direction and magnitude of high-suitability changes differed among projections, underscoring the importance of explicitly representing forecasting uncertainty across climate models and emission pathways [55]. Future studies should assess whether elevational gradients and fine-scale environmental buffering can maintain locally suitable conditions, particularly in Madeira and the Canary Islands, where the greatest losses were projected [56,57].

The two Portuguese archipelagos present contrasting outlooks. The Azores retain extensive climatic suitability for both species, although shifts among suitability classes indicate changes in relative suitability rather than complete climatic stability. Their wetter, strongly oceanic conditions may favor the continued availability of suitable macroclimates [27,52], although local persistence will also depend on shaded and microclimatically buffered habitats [43,44,58]. Cave entrances and other azonal environments can provide high humidity, low thermal variability, and reduced irradiance. In fact, *T. fontanum* has been reported as one of the most abundant bryophytes at Azorean cave entrances [36,46,59,60]. In Madeira, both species occur in humid laurel forests, and *T. virens* can be locally frequent. Nevertheless, the projected loss of climatic suitability, particularly of highly suitable areas, raises concerns about their future conservation [43,44,61].

Spanish populations are among the most vulnerable components of the *Tetrastichium* range. Although new localities have improved knowledge of both species in the Canary Islands [62], most populations remain small, isolated, and confined to scarce azonal or microclimatically buffered habitats such as caves, increasing their vulnerability to hydrological alteration and local disturbance [43,44]. Climatic suitability is already highly restricted in the archipelago, and no cells remained above the 0.25 threshold for either species under the most adverse UKESM1-0-LL projections by the end of the century. In mainland Spain, *T. fontanum* is known from a single locality [63], whereas *T. virens* is represented by an extremely small, restricted, and fragmented population [64]. Although the Iberian Peninsula was excluded from the projections, the isolation of these populations suggests limited resilience to drying, hydrological alteration, and habitat degradation.

These geographical contrasts raise questions about whether current conservation assessments adequately reflect the vulnerability of both species to climate change. *Tetrastichium fontanum* is classified as Vulnerable, whereas *T. virens* is Near Threatened [34]. However, *T. virens* is scarce in the Canary Islands, relatively frequent in Madeira, and sporadic in the Azores [44,65]. Its restricted distribution in parts of its range, small and isolated populations, and projected losses of climatic suitability in Madeira and the Canary Islands warrant reassessment of its conservation status. Criterion A3c may be relevant to both species because it allows projected population reductions to be assessed using declines in area of occupancy, extent of occurrence, and/or habitat quality [47,66]. Formal application would nevertheless require evidence of population reduction over the appropriate assessment period and updated data on population size and trends, generation length, area of occupancy, extent of occurrence, habitat condition, connectivity, and the relationship between climatic suitability and mature individuals.

These findings provide guidance for surveys and conservation management. Areas projected to retain climatic suitability beyond known localities may be priority targets for field surveys, whereas known populations in Madeira and the Canary Islands may warrant particular monitoring. Projected gains, especially in the Azores, represent climatic opportunities rather than expected range expansion because geographical isolation, limited propagule availability, habitat discontinuity, and establishment constraints may prevent bryophytes from tracking climate change [28]. Field validation is therefore needed to assess substrate availability, forest structure, hydrology, microhabitats, and propagule sources [5,67]. Conservation should prioritize known populations and macroclimatically suitable, locally buffered habitats [24,37] by preserving mature laurel and heath forests, canopy cover, and hydrology while limiting water abstraction, drainage, fire, trampling, and canopy opening in ravines, springs, cave entrances, and lava tubes [36,58].

Several limitations should be considered. Correlative species-distribution models (SDMs) represent hypotheses of environmental suitability rather than realized distributions because occurrence also depends on dispersal, biotic interactions, land-use history, sampling bias, substrate availability, and microhabitat conditions [1,5]. Macroclimate-based predictions may not capture the fine-scale climatic buffering and steep environmental gradients of Macaronesian islands [37,68,69], and the two selected GCMs represent contrasting climate sensitivities but not the full range of inter-model uncertainty. Predictor selection preceded spatial cross-validation, so some information leakage and slightly optimistic estimates of model performance cannot be excluded. Although variability among ten pseudo-absence samples was represented, sensitivity to alternative exclusion distances was not formally evaluated. The models also omit dispersal, establishment, demographic processes, and future habitat change [16,70], which is relevant because bryophytes may lag behind shifting climatic conditions despite their high dispersal capacities [28]. Consequently, the projections represent broad-scale patterns of climatic suitability rather than precise predictions of local occurrence or deterministic forecasts of population decline or extinction.

## 4. Materials and Methods

### 4.1. Study Area

The study focused on the three main Macaronesian archipelagos: the Azores, Madeira, and the Canary Islands. These oceanic archipelagos exhibit pronounced latitudinal, elevational, and climatic gradients. Differences in geological history, trade-wind exposure, topography, and precipitation regimes produce marked environmental heterogeneity over short distances. *Tetrastichium* populations are primarily associated with humid, thermally buffered habitats, including laurel and heath forests, ravines, springs, cave entrances, and shaded rock faces [43,44]. Although *T. fontanum* and *T. virens* also occur in the southwestern Iberian Peninsula and their occurrences were included in model calibration, continental predictions were excluded from the final area summaries and overlap analyses because MESS filtering retained only a limited continental area with climatic conditions analogous to those used as the reference for projection.

### 4.2. Occurrence Data

**Figure 9:**
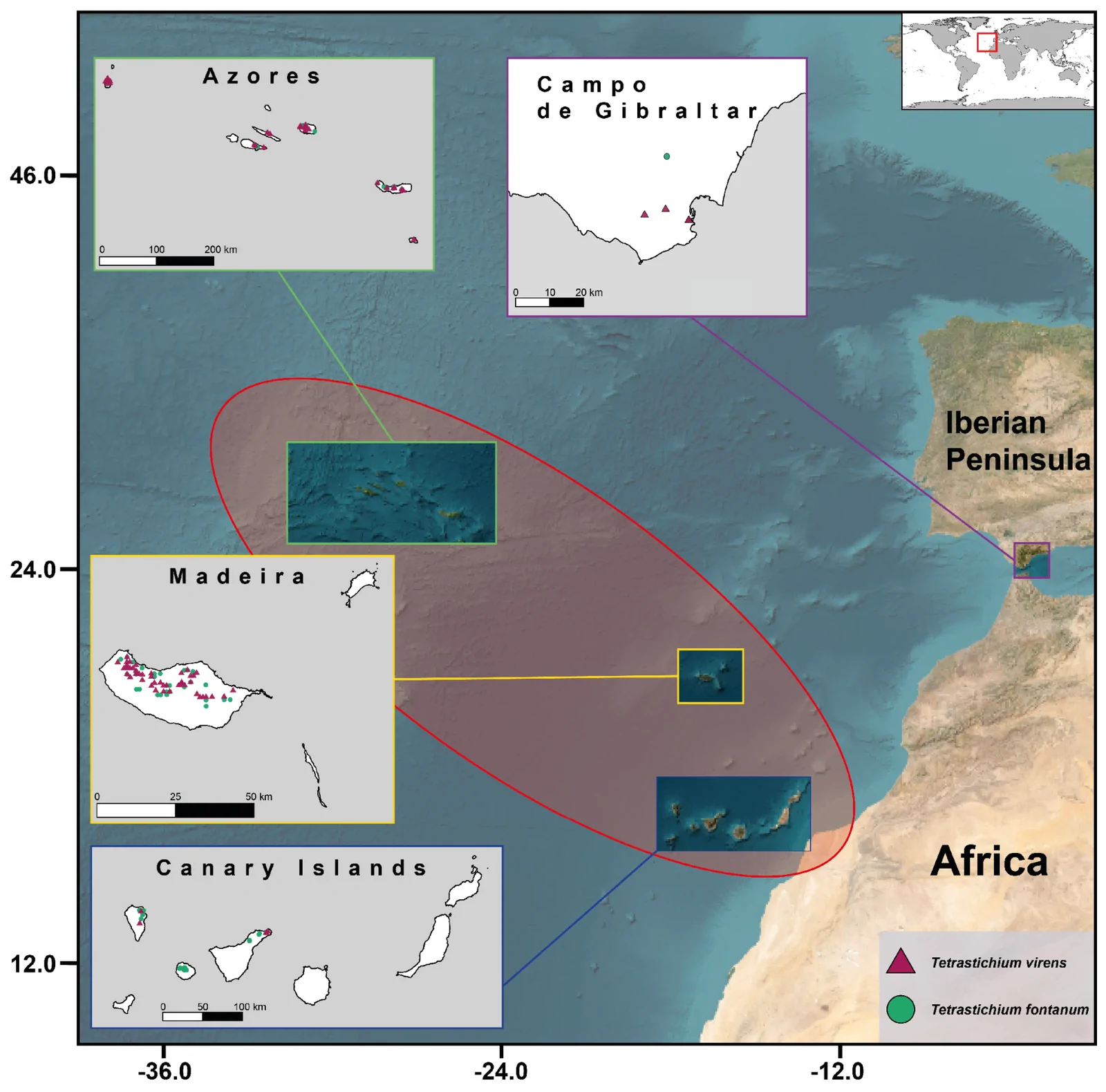
Geographical setting and occurrence records of *T. fontanum* and *T. virens* in the Ibero-Macaronesian region. The red frame in the upper-right inset indicates the location of the Ibero-Macaronesian region within the global context. The main map shows the location of the study areas in the northeastern Atlantic, including the Azores, Madeira and Canary Islands, and the Campo de Gibraltar. The shaded red ellipse highlights the Macaronesian sector. *T. fontanum* is represented by green circles and *T. virens* by magenta triangles.

Occurrence records were obtained from GBIF [71,72] and complemented with original records collected by the authors across the three Macaronesian archipelagos. From GBIF, we retained only georeferenced presence records based on preserved specimens and standardized species names by removing leading, trailing, and duplicate spaces. Records lacking coordinates or exceeding the maximum positional uncertainty compatible with the 1 km spatial resolution (approximately 707 m, corresponding to half the diagonal of a 1 km grid cell) were excluded. We also screened each coordinate pair to identify duplicates and potentially erroneous locations, including equal or zero longitude–latitude values. The resulting 147 GBIF records, 45 for *T. fontanum* and 102 for *T. virens*, were combined with 125 original records, comprising 89 records of *T. fontanum* and 36 of *T. virens*. To identify spatial duplicates between and within both data sources, all records were assigned to a common 1 km^2^ grid and only one occurrence per occupied cell was retained. This procedure also reduced sampling bias and spatial autocorrelation, resulting in a final dataset of 180 occurrence records, with 90 records for each species (Figure 9).

### 4.3. Climate Data

We retrieved the 19 bioclimatic variables from WorldClim 2.1 using the geodata R package [73]. WorldClim has provided high-spatial-resolution global weather and climate data since 2005 (version 1.4 [74]) and has been progressively improved, including in version 2.1 [25]. The dataset consists of spatially interpolated monthly climate data, with downscaled temperature and precipitation among its most widely used variables. The WorldClim2 baseline climate data were created using thin-plate spline interpolation with covariates such as elevation, distance to the coast, and variables derived from MODIS (Moderate Resolution Imaging Spectroradiometer), including maximum and minimum land surface temperature and cloud cover. The interpolation was performed for 23 separate regions delineated by station density. The baseline period for WorldClim2 is 1970–2000, and the resolution employed here is 30 arc seconds (~1 km at the equator). Future WorldClim2 data include projections of monthly values from multiple Global Climate Models (GCMs) for the four SSPs, downscaled and calibrated using the WorldClim2 baseline dataset. There is minimal information on the downscaling of WorldClim2 future climate projections, as these are described only as relative and absolute deltas between baseline and future GCM runs, spatially interpolated to 1 km resolution.

To project future climatic suitability, we selected two GCMs from the Coupled Model Intercomparison Project 6 (CMIP6) based on their Equilibrium Climate Sensitivity (ECS), a measure of how climate models respond to a doubling of atmospheric carbon dioxide. We selected the less-sensitive MPI-ESM1-2-HR (ECS = 2.98) and the more-sensitive UKESM1-0-LL (ECS = 5.34) [75]. For each GCM, we considered the SSP3-7.0 and SSP5-8.5 scenarios for the periods 2061–2080 and 2081–2100.

Variable selection was performed using the collinear R package [76], an integrative framework for selecting predictors while controlling for multicollinearity. Predictor variables were first ranked by discriminatory performance using univariate models (Pres (0, 1) ~ Predictor), as measured by the Area Under the Receiver Operating Characteristic Curve (AUC). Starting with the variable with the highest AUC, additional predictors were retained only if they met predefined thresholds for collinearity: Pearson’s correlation coefficient |r| < 0.7 and variance inflation factor (VIF) < 5. This procedure identified a subset of predictors with high discriminatory power while minimizing redundancy. Accordingly, mean diurnal range (BIO2), maximum temperature of the warmest month (BIO5), and precipitation of the wettest quarter (BIO16) were selected for *T. fontanum*, whereas mean diurnal range (BIO2), maximum temperature of the warmest month (BIO5), and precipitation of the coldest quarter (BIO19) were selected for *T. virens*. Predictor selection was performed once using the complete calibration dataset, and the resulting species-specific predictor sets were subsequently used in all spatial cross-validation folds.

### 4.4. Modeling Framework

We fitted Boosted Regression Trees (BRTs) to model the climatic suitability of both species. Models were fitted using the gbm.step function from the dismo package, which relies on the gbm package [77], and spatial predictions were generated using the predict function from the terra package [78]. BRT is an ensemble method in which regression trees are added sequentially, with each additional tree reducing the predictive error of the existing ensemble and its contribution scaled by the learning rate [79]. This approach can accommodate nonlinear relationships and interactions among predictors. Tree complexity (5), learning rate (0.01), and bag fraction (0.5) were fixed a priori. The optimal number of trees was determined independently for each model using the internal 10-fold cross-validation implemented in gbm.step, which was used exclusively for tree-number selection and was distinct from the subsequent spatial cross-validation used for model evaluation. Starting with 50 trees, additional trees were added in increments of 50, up to a maximum of 10,000, and the number minimizing mean cross-validation deviance was retained [53].

Because both species occur in Macaronesia and the southwestern Iberian Peninsula, all filtered occurrence records from both regions were included when delineating the calibration area and fitting the models. We delineated the calibration area using the getRegion clustering approach implemented in the fuzzySim R package [80], which groups occurrence records according to their geographical affinities while weighting the best-represented regions. We generated ten independently sampled pseudo-absence datasets within the calibration area using the selectAbsences function in the fuzzySim R package. For each dataset, an initial pool of ten candidate pseudo-absences per presence was generated. Distances from known occurrences were calculated from WGS84 coordinates using the haversine formula. Candidate pseudo-absences located within 800 m of a known presence were excluded to reduce the likelihood of assigning pseudo-absences to the immediate surroundings of occurrence records at the approximately 1 km spatial resolution used. A random subset of the remaining candidates was then selected to obtain the same number of presences and pseudo-absences, resulting in a sample prevalence of 0.5, as recommended by Barbet-Massin et al. [81] for machine learning techniques. A fixed random seed (432) was used to ensure reproducibility. One BRT model replicate was fitted to each of the ten pseudo-absence datasets. Each model replicate was evaluated using spatial 10-fold cross-validation, resulting in 100 fitted cross-validation models per species [12]. Following Sillero and Barbosa [67], the ten replicate predictions were averaged to produce the final suitability map, while their pixel-level standard deviation was calculated to represent variability associated with pseudo-absence sampling and model fitting. Variable contributions were also calculated for each replicate. Model evaluation was based on five complementary metrics. The area under the receiver operating characteristic curve (AUC), standardized and threshold-dependent true skill statistic (sTSS), and sensitivity were used to assess discrimination between presences and pseudo-absences. The Continuous Boyce Index was used to evaluate the correspondence between predicted suitability and the distribution of presences, while Nagelkerke’s pseudo-R^2^ quantified improvement in model fit relative to a null model [82]. Comparisons between fitted and null models have been recommended for modeling bryophytes and other species with small sample sizes [83]. All the aforementioned metrics were calculated using the modEvA R package [84].

Projecting models into areas or time periods where environmental conditions are not analogous to those prevailing within the calibration area may yield unreliable predictions. We therefore identified which areas of Macaronesia are projected to retain analogous climates in 2061–2080 and 2081–2100 relative to the current climate, and which continental areas currently exhibit, or are projected to exhibit during the same periods, climatic conditions analogous to those currently found in Macaronesia, using multivariate environmental similarity surfaces (MESS) [85]. MESS measures the environmental similarity of each projected pixel to the reference range defined for the selected predictor variables. A positive value indicates that the pixel lies within this reference range, whereas a negative value indicates that at least one predictor falls outside it. Areas predicted as suitable but located outside the range of analogous climates were therefore excluded as extrapolation regions. Given the extensive climatic extrapolation identified by the MESS analyses across most of the Iberian Peninsula, projections for this region were excluded from subsequent analyses. For this purpose, we employed the mess function from the predicts R package [86]. Subsequently, each prediction was filtered using its corresponding MESS output, retaining only cells with MESS values greater than zero.

For descriptive mapping and area summaries, continuous suitability predictions were divided into four equal-width categories: unsuitable (0–0.25), low suitability (0.25–0.50), moderate suitability (0.50–0.75), and high suitability (0.75–1.00). Because threshold selection can substantially influence estimates of suitable area [87], these intervals were treated as descriptive climatic-suitability classes rather than as independently validated presence–absence or habitat thresholds. Changes in their extent therefore represent changes in predicted climatic suitability and not demonstrated changes in realized habitat availability. Current suitability maps were also used to identify climatically suitable areas without confirmed records, which may be priorities for future field surveys. The entire geoprocessing workflow was carried out using the terra R package [78].

### 4.5. Climatic-Suitability Overlap

To quantify changes in the spatial distribution of climatic suitability between current and future predictions, we computed pairwise overlap using Schoener’s D [88]. Before calculation, suitability values were normalized to sum to one across the cells being compared. The metric ranges from 0, indicating no overlap, to 1, indicating identical normalized spatial patterns. Higher values therefore indicate greater spatial correspondence in relative climatic suitability, whereas lower values indicate greater spatial redistribution. Schoener’s D does not directly quantify absolute changes in suitability, habitat loss, population decline, or extinction risk. Values were calculated using the calc.niche.overlap function in the ENMeval R package [89].

## 5. Conclusions

This study provides the first regional assessment of the current and future climatic suitability of *Tetrastichium fontanum* and *T. virens* across Macaronesia. The projections reveal contrasting responses between species and among archipelagos, with climatic suitability generally retained or increased in the Azores, particularly for *T. virens*, but substantially reduced in Madeira and the Canary Islands. For *T. virens*, the combination of its geographically discontinuous distribution, small and isolated populations in parts of its range, and projected contractions in climatic suitability in Madeira and the Canary Islands suggests that its current Near Threatened status warrants reassessment using updated information on population size and trends, distribution, habitat condition, and connectivity. Conservation efforts should prioritize extant populations and the maintenance of humid, microclimatically buffered habitats, particularly in areas where climatic suitability is projected to decline. Integrating these climatic projections with population monitoring and field-based assessments of habitat condition will provide a stronger basis for developing effective conservation strategies for both species across Macaronesia.

## Figures and Tables

**Table 1:** Performance metrics of the Boosted Regression Tree (BRT) models for *Tetrastichium fontanum* and *Tetrastichium virens*. Values correspond to the mean and standard deviation (±SD) calculated across the ten model replicates. Model discrimination was assessed using the Area Under the Receiver Operating Characteristic Curve (AUC), standardized True Skill Statistic (sTSS), and sensitivity. Model performance was additionally assessed using the Continuous Boyce Index and Nagelkerke’s pseudo-R^2^, the latter representing improvement in model fit relative to a null model.

Metric	Species	Mean ± SD
AUC ROC	*T. fontanum*	0.88 ± 0.03
	*T. virens*	0.88 ± 0.03
Boyce	*T. fontanum*	0.68 ± 0.19
	*T. virens*	0.70 ± 0.20
Nagelkerke’s Pseudo-R^2^	*T. fontanum*	0.57 ± 0.06
	*T. virens*	0.57 ± 0.06
Sensitivity	*T. fontanum*	0.95 ± 0.05
	*T. virens*	0.94 ± 0.06
sTSS	*T. fontanum*	0.81 ± 0.03
	*T. virens*	0.81 ± 0.03

**Table 2:** Current and projected climatic suitability of *Tetrastichium fontanum* across the Azores, Madeira, and the Canary Islands under the MPI-ESM1-2-HR and UKESM1-0-LL global climate models, the SSP3-7.0 and SSP5-8.5 pathways, and the 2061–2080 and 2081–2100 periods. Values represent the percentage of the terrestrial area of each archipelago classified as having low (0.25–0.50), moderate (0.50–0.75), or high (0.75–1.00) climatic suitability. Changes relative to current conditions (Δ) are expressed in percentage points, with positive values indicating gains and negative values indicating losses, as represented by green and red cell shading, respectively; darker shades indicate larger absolute changes.

*Tetrastichium fontanum*									
				Suitability Class (%)			Change Relative to Current (Percentage Points)		
Archipelago	Model	SSP	Period	Low	Moderate	High	Δ Low	Δ Moderate	Δ High
**Azores**	Current	–	–	28.4	22.8	34.9	–	–	–
	MPI	SSP370	2061–2080	34.5	32.5	23.1	+6.1	+9.7	−11.8
			2081–2100	32.4	31.8	28.6	+4.0	+9.0	−6.3
		SSP585	2061–2080	33.2	31.3	28.6	+4.8	+8.5	−6.3
			2081–2100	32.4	29.7	28.1	+4.0	+6.9	−6.8
	UKESM	SSP370	2061–2080	30.3	28.1	39.0	+1.9	+5.3	+4.1
			2081–2100	29.2	27.1	41.6	+0.8	+4.3	+6.7
		SSP585	2061–2080	28.8	29.2	37.9	+0.4	+6.4	+3.0
			2081–2100	30.1	26.1	41.5	+1.7	+3.3	+6.6
**Madeira**	Current	–	–	14.4	16.8	43.7	–	–	–
	MPI	SSP370	2061–2080	18.1	17.1	26.1	+3.7	+0.3	−17.6
			2081–2100	18.3	14.1	20.9	+3.9	−2.7	−22.8
		SSP585	2061–2080	16.3	16.5	24.8	+1.9	−0.3	−18.9
			2081–2100	24.1	12.6	17.5	+9.7	−4.2	−26.2
	UKESM	SSP370	2061–2080	23.8	6.3	12.9	+9.4	−10.5	−30.8
			2081–2100	22.4	5.1	3.6	+8.0	−11.7	−40.1
		SSP585	2061–2080	26.8	4.5	5.7	+12.4	−12.3	−38.0
			2081–2100	19.3	0.6	0.3	+4.9	−16.2	−43.4
**Canary Islands**	Current	–	–	3.3	2.8	3.8	–	–	–
	MPI	SSP370	2061–2080	1.1	1.0	2.2	−2.2	−1.8	−1.6
			2081–2100	0.9	0.9	1.3	−2.4	−1.9	−2.5
		SSP585	2061–2080	0.8	1.0	1.7	−2.5	−1.8	−2.1
			2081–2100	0.7	0.6	0.7	−2.6	−2.2	−3.1
	UKESM	SSP370	2061–2080	0.3	0.2	0.0	−3.0	−2.6	−3.8
			2081–2100	0.0	0.0	0.0	−3.3	−2.8	−3.8
		SSP585	2061–2080	0.2	0.0	0.0	−3.1	−2.8	−3.8
			2081–2100	0.0	0.0	0.0	−3.3	−2.8	−3.8

**Table 3:** Current and projected climatic suitability of *Tetrastichium virens* across the Azores, Madeira, and the Canary Islands under the MPI-ESM1-2-HR and UKESM1-0-LL global climate models, the SSP3-7.0 and SSP5-8.5 pathways, and the 2061–2080 and 2081–2100 periods. Values represent the percentage of the terrestrial area of each archipelago classified as having low (0.25–0.50), moderate (0.50–0.75), or high (0.75–1.00) climatic suitability. Changes relative to current conditions (Δ) are expressed in percentage points, with positive values indicating gains and negative values indicating losses, as represented by green and red cell shading, respectively; darker shades indicate larger absolute changes.

*Tetrastichium virens*									
				Suitability Class (%)			Change Relative to Current (Percentage Points)		
Archipelago	Model	SSP	Period	Low	Moderate	High	Δ Low	Δ Moderate	Δ High
**Azores**	Current	–	–	47.7	44.7	5.9	–	–	–
	MPI	SSP370	2061–2080	25.8	55.3	18.1	−21.9	+10.6	+12.2
			2081–2100	23.3	53.2	23.0	−24.4	+8.5	+17.1
		SSP585	2061–2080	24.8	52.1	22.2	−22.9	+7.4	+16.3
			2081–2100	28.9	44.6	24.8	−18.8	−0.1	+18.9
	UKESM	SSP370	2061–2080	18.2	45.5	36.3	−29.5	+0.8	+30.4
			2081–2100	19.4	43.7	36.8	−28.3	−1.0	+30.9
		SSP585	2061–2080	18.1	43.3	38.6	−29.6	−1.4	+32.7
			2081–2100	20.9	43.2	35.3	−26.8	−1.5	+29.4
**Madeira**	Current	–	–	11.9	22.7	39.5	–	–	–
	MPI	SSP370	2061–2080	22.1	17.4	26.3	+10.2	−5.3	−13.2
			2081–2100	25.7	16.8	19.9	+13.8	−5.9	−19.6
		SSP585	2061–2080	23.7	13.8	24.4	+11.8	−8.9	−15.1
			2081–2100	28.9	11.3	17.3	+17.0	−11.4	−22.2
	UKESM	SSP370	2061–2080	33.9	9.6	4.1	+22.0	−13.1	−35.4
			2081–2100	16.7	3.8	0.8	+4.8	−18.9	−38.7
		SSP585	2061–2080	30.2	9.2	2.3	+18.3	−13.5	−37.2
			2081–2100	8.7	1.2	0.0	−3.2	−21.5	−39.5
**Canary Islands**	Current	–	–	3.9	2.1	0.0	–	–	–
	MPI	SSP370	2061–2080	1.3	0.2	0.0	−2.6	−1.9	0.0
			2081–2100	0.6	0.0	0.0	−3.3	−2.1	0.0
		SSP585	2061–2080	1.0	0.1	0.0	−2.9	−2.0	0.0
			2081–2100	0.4	0.0	0.0	−3.5	−2.1	0.0
	UKESM	SSP370	2061–2080	0.1	0.0	0.0	−3.8	−2.1	0.0
			2081–2100	0.0	0.0	0.0	−3.9	−2.1	0.0
		SSP585	2061–2080	0.0	0.0	0.0	−3.9	−2.1	0.0
			2081–2100	0.0	0.0	0.0	−3.9	−2.1	0.0

## Data Availability

The MESS-derived vector masks, predictor-response curves, and pixel-level prediction-variability maps and GeoTIFF rasters generated in this study are openly available in Figshare at https://doi.org/10.6084/m9.figshare.33426271.
